# Impact of Radiotherapy on the Condition of the Oral Cavity in Patients with Head and Neck Cancer

**DOI:** 10.3390/jcm15135107

**Published:** 2026-06-30

**Authors:** Tilen Dervarič, Elias Pascal Bračko, Petra Povalej Brzan, Janez Rebol

**Affiliations:** 1 Faculty of Medicine, University of Maribor, SI-2000 Maribor, Slovenia; dervarictilen@gmail.com (T.D.); eliasbracko11@gmail.com (E.P.B.); petra.povalej@um.si (P.P.B.); 2 Clinic for Otorhinolaryngology and Head and Neck Surgery, University Medical Centre Maribor, SI-2000 Maribor, Slovenia

**Keywords:** head and neck cancer, radiotherapy, oral complications, xerostomia, dysphagia, dental status, trismus, EORTC QLQ-C30, EORTC QLQ-HN43, quality of life

## Abstract

**Background/Objectives:** Radiotherapy (RT) for head and neck cancer (HNC) is associated with oral and functional toxicity, but integrated data combining dental status, nutritional indicators and patient-reported outcomes remain limited. This study evaluated oral, dental, functional, nutritional and quality-of-life outcomes after RT. **Methods:** This exploratory observational study combined a retrospective record review with a prospective cross-sectional assessment of patient-reported outcomes and included 20 adults treated with RT for HNC in a regional Slovenian clinical setting. Clinical, oncological, dental and nutritional variables were extracted from records, while patient-reported outcomes were assessed once at post-treatment follow-up using the EORTC QLQ-C30, EORTC QLQ-HN43 and a focused oral-health questionnaire. **Results:** The median time from RT completion to questionnaire assessment was 3.0 months. Dysphagia, xerostomia and taste disturbances after treatment were recorded in 85.0%, 80.0% and 75.0% of patients, respectively. Trismus was present in 40.0%, and weight loss >10% in 55.0%. QLQ-HN43 domains with the highest burden included fear of progression, dry mouth/sticky saliva, problems opening the mouth, social eating, weight loss and teeth problems. Swallowing was strongly associated with social eating (rho = 0.798, *p* < 0.001), and mouth opening was inversely associated with patient-reported problems opening the mouth (rho = −0.610, *p* = 0.004). **Conclusions:** In this small exploratory cohort, substantial early post-treatment oral-functional morbidity was observed. The findings suggest the potential value of integrated survivorship follow-up incorporating dental, nutritional, functional, and patient-reported outcome monitoring.

## 1. Introduction

Head and neck cancer (HNC) comprises a heterogeneous group of malignancies arising from the upper aerodigestive tract, including cancers of the lips, oral cavity, pharynx, larynx, nasal cavity, paranasal sinuses, salivary glands, skin of the head and neck region, and cancers of unknown primary presenting with cervical lymph node metastases [1,2]. Squamous cell carcinoma accounts for more than 90% of cases [3]. In 2020, nearly one million new HNC cases and approximately 467,000 deaths were estimated worldwide, representing a substantial global oncological burden [4]. Although tobacco and alcohol remain major established risk factors, the epidemiology of HNC has changed in recent decades, particularly due to the increasing incidence of human papillomavirus (HPV)-associated oropharyngeal cancer [5,6,7].

Treatment of HNC is frequently multimodal and may include surgery, radiotherapy (RT), chemotherapy, targeted therapy, or combinations of these modalities. Treatment selection depends on tumor site, stage, resectability, expected functional outcomes, comorbidities, and the balance between disease control and treatment-related toxicity [8,9,10]. Radiotherapy is a key component of both definitive and adjuvant treatment. Modern RT techniques allow improved dose conformity and better sparing of organs at risk, including the salivary glands [11,12,13]. Nevertheless, improvements in treatment planning do not eliminate radiation-related toxicity, particularly when radiosensitive oral and oropharyngeal tissues are located within or near the irradiated volume [14,15].

Oral and oropharyngeal complications are among the most clinically relevant adverse effects of RT and chemoradiotherapy in patients with HNC. Radiation-related toxicity is commonly classified as acute, occurring during treatment or within the early post-treatment period, and chronic, developing later and potentially persisting for years [14,15]. Acute complications include oral mucositis, mucosal infections, pain, dysgeusia, and dysphagia [15,16,17]. Dysgeusia may affect a substantial proportion of patients after RT or chemoradiotherapy and can persist beyond the active treatment period [18]. Chronic sequelae may include persistent salivary gland dysfunction, xerostomia, tissue fibrosis, trismus, neuropathic pain, radiation-related dental disease, periodontal deterioration, tooth loss, and osteoradionecrosis [14,15,19]. The combination of RT with chemotherapy may further increase the frequency and severity of treatment-related toxicity [20].

These complications are not limited to local oral discomfort but may substantially affect essential functions such as chewing, swallowing, speech, oral hygiene, nutrition, and social eating. Dysgeusia and mucosal pain may negatively influence food intake and quality of life [16,18]. Radiation-induced fibrosis of the tongue, pharyngeal muscles, masticatory muscles, and temporomandibular joint may contribute to dysphagia and trismus [21,22,23]. Xerostomia and salivary hypofunction impair lubrication, buffering, remineralization, and oral clearance, thereby increasing the risk of rapidly progressive radiation-related caries and subsequent extractions [19,24,25]. Periodontal deterioration after RT may be promoted by hyposalivation, microbiological changes, and accelerated alveolar bone loss [26,27,28]. Consequently, oral sequelae after HNC treatment may affect not only local comfort but also eating, swallowing, oral function, and health-related quality of life [16,21,29].

Dental management is therefore an integral component of multidisciplinary care for patients undergoing RT for HNC. Early dental assessment, elimination of oral foci, preventive counseling, fluoride therapy, oral hygiene instruction, monitoring of mucosal and salivary changes, trismus prevention, and long-term dental follow-up are recommended to reduce the risk of post-radiation complications [30,31,32]. Patients with HNC often present with poorer oral health even before RT, including a high burden of caries, periodontal disease, retained roots, and high DMFT scores [33,34,35]. This supports the need for individualized dental protocols before, during, and after oncological treatment [31,36,37]. After RT, patients remain at high risk for radiation-related caries and osteoradionecrosis, particularly when invasive dentoalveolar procedures are required in irradiated bone [37,38,39].

Prevention and management of oral mucositis are important components of supportive care during cancer treatment. Contemporary preventive approaches include patient education, professional and home oral care, anti-inflammatory agents such as benzydamine, photobiomodulation, cryotherapy, mucosal-protective agents, analgesic mouth rinses, and selected nutritional or pharmacological interventions. Because the available evidence and clinical indications vary among these modalities, their use should be adapted to the specific oncological treatment setting and individual patient characteristics [40].

Although the oral complications of RT in HNC are well recognized [14,15], there remains a need for studies that integrate pre- and post-radiotherapy oral and dental status, treatment-related adverse effects, nutritional indicators, and patient-reported outcomes obtained at post-treatment follow-up using validated quality-of-life instruments. In particular, combining clinical data with EORTC QLQ-C30, the HNC-specific EORTC QLQ-HN43 module, and focused oral-health questionnaires may provide a more comprehensive understanding of the oral-functional burden experienced by patients after treatment.

The specific knowledge gap addressed by this study is the limited availability of real-world data that simultaneously characterize clinical oral status, dentition, nutritional change, objective mouth opening, and validated patient-reported outcomes within the same post-treatment cohort. By integrating these domains, including preventive and supportive dental-care practices, the present study provides a multidimensional description of early post-radiotherapy oral-functional burden that cannot be captured by any single clinical or quality-of-life measure. Therefore, this observational study aimed to compare oral and dental status before radiotherapy and at post-treatment follow-up in patients with HNC, and to evaluate the occurrence of oral and functional adverse effects after radiotherapy. Secondary aims were to assess nutritional status, including body mass index and weight loss, to evaluate post-treatment health-related quality of life using EORTC QLQ-C30 and EORTC QLQ-HN43, and to explore associations between xerostomia, dysphagia, taste disturbances, trismus, dental status, nutritional indicators, patient-reported oral burden, and quality-of-life outcomes. In addition, the study aimed to describe patient-reported use of preventive and supportive dental measures.

## 2. Materials and Methods

### 2.1. Study Design, Setting, and Participants

This observational study combined a retrospective review of existing clinical, oncological, dental, and nutritional records with a prospective cross-sectional assessment of patient-reported outcomes during a single post-treatment follow-up visit. The timeframe for determining clinical eligibility and conducting follow-up assessments spanned from 1 May 2025, to 31 January 2026. After approval by the relevant ethics committees, pre-existing patient data generated between 1 May 2025, and 20 October 2025, were retrospectively reviewed. Patient enrollment, informed consent, and questionnaire administration were conducted between 21 October 2025, and 31 January 2026. The study was conducted at the University Medical Centre Maribor, in collaboration with the Community Health Centre dr. Adolfa Drolca Maribor, Slovenia. Eligible patients were adults diagnosed with head and neck cancer who had received radiotherapy to the head and neck region as part of their oncological treatment, irrespective of whether radiotherapy was delivered as definitive treatment, adjuvant treatment, or in combination with surgery and/or systemic therapy.

The inclusion criteria were: diagnosis of head and neck cancer, treatment with radiotherapy to the head and neck region, age ≥ 18 years, available medical and dental documentation, and completion of patient-reported outcome questionnaires at follow-up. Patients were excluded if key clinical data or patient-reported outcome data were missing to an extent that prevented calculation of the main outcome variables.

A total of 24 patients were initially identified for the study. Four patients were excluded because patient-reported outcome data could not be obtained, as they were unable to attend the follow-up assessment and complete the questionnaires. Reasons included death before questionnaire assessment, severe clinical deterioration, or poor general condition preventing participation. The final study cohort consisted of 20 patients.

The primary objectives were to compare oral and dental status before radiotherapy and at post-treatment follow-up, and to determine the frequency of oral and functional adverse effects after radiotherapy. Secondary objectives were to assess nutritional status, body mass index, weight loss, health-related quality of life, and associations between treatment-related oral complications, dental status, nutritional indicators, patient-reported oral burden, and EORTC QLQ-C30/QLQ-HN43 outcomes.

### 2.2. Clinical, Oncological, Dental, and Nutritional Data

A retrospective review of medical and dental records was performed. The following demographic and oncological variables were collected: age, sex, diagnosis, tumor site, histopathological diagnosis, TNM classification, HPV status, tumor side, treatment modality, radiotherapy dose, number of radiotherapy fractions, chemotherapy status, chemotherapy regimen, number of chemotherapy cycles, surgery status, date of radiotherapy completion, and date of questionnaire completion. Time since radiotherapy was calculated as the interval between radiotherapy completion and questionnaire completion and expressed in months.

Treatment-related oral and functional complications were recorded from clinical documentation and follow-up assessment, including oral mucositis, radiodermatitis, dysphagia, xerostomia, taste disturbances, and trismus. When available, severity grades were recorded separately for complications occurring during treatment and after treatment.

Dental variables included periodontitis status, periodontitis stage and grade, number of teeth before treatment, change in the number of teeth from pre-radiotherapy assessment to follow-up, and edentulism after treatment. The change in the number of teeth was calculated as the difference between the number of teeth before treatment and the number of teeth at post-treatment follow-up.

Clinical examination included measurement of body weight and height, assessment of maximum mouth opening, and recording of the number of remaining teeth. Maximum mouth opening was measured in millimeters as the maximum interincisal distance in dentate patients; in edentulous patients, the distance between the opposing alveolar ridges was recorded. A binary trismus variable was derived using a threshold of ≤35 mm, while the continuous mouth-opening measurement was retained for correlation analyses.

Nutritional variables included body weight before radiotherapy, current body weight at follow-up, absolute and percentage weight loss, body mass index before radiotherapy, and body mass index at follow-up. Body mass index was calculated as weight in kilograms divided by height in meters squared. Percentage weight loss was calculated as the difference between the weight before radiotherapy and the current weight, divided by the weight before radiotherapy and multiplied by 100. A binary variable for clinically relevant weight loss was created using a threshold of >10% weight loss.

### 2.3. Patient-Reported Outcome Measures

Patient-reported outcomes were assessed once during a routine post-treatment follow-up visit. The questionnaires were not administered immediately after radiotherapy in all patients; therefore, the date of radiotherapy completion and the date of questionnaire completion were recorded for each participant. Questionnaires were self-administered, with assistance provided by the investigators when required.

Health-related quality of life was assessed using the validated Slovenian version of the EORTC QLQ-C30 questionnaire [41]. The following scales and single-item measures were calculated: global health status/quality of life, physical functioning, role functioning, emotional functioning, cognitive functioning, social functioning, fatigue, nausea and vomiting, pain, dyspnea, insomnia, appetite loss, constipation, diarrhea, and financial difficulties. All QLQ-C30 scales were linearly transformed to a 0–100 scale according to EORTC scoring procedures [42]. For global health status and functional scales, higher scores indicate better functioning or better quality of life, whereas higher symptom scores indicate greater symptom burden.

Head and neck cancer-specific symptoms and functional problems were assessed using the validated Slovenian version of the EORTC QLQ-HN43 module [43]. Calculated scales and single-item measures included pain in the mouth, swallowing, problems with teeth, dry mouth and sticky saliva, problems with senses, speech, body image, social eating, sexuality, shoulder problems, skin problems, fear of progression, problems opening the mouth, coughing, social contact, swelling in the neck, weight loss, wound healing problems, and neurological problems. All QLQ-HN43 scores were transformed to a 0–100 scale according to EORTC scoring procedures [42], with higher scores indicating greater symptom burden or more problems.

A study-specific oral-health questionnaire was used to assess patient-reported oral problems, symptom severity, functional impact, preventive and supportive measures, and subjective perception of oral health after treatment. The questionnaire recorded the occurrence and severity of dry mouth, oral pain, burning or irritating sensation, chewing difficulties, swallowing difficulties, taste disturbances, limited mouth opening, ulcers or mucosal inflammation, and oral fungal infection. Severity and functional impact responses were dichotomized as not at all/a little versus quite a bit/very much.

Several summary variables were derived from the questionnaire: oral symptom count, severe symptom count, functional impact count, and preventive/supportive care score. The preventive/supportive care score included reported measures such as dental examination before treatment, oral hygiene instructions, fluoride use, saliva substitutes or products for dry mouth, mouth-opening exercises, regular dental follow-up, and perceived adequacy of information. Difficult access to dental care was not included in this score because it represents a barrier rather than a preventive measure.

### 2.4. Outcomes

The primary descriptive outcomes were oral and dental status before radiotherapy and at post-treatment follow-up, and the frequency of oral and functional adverse effects after radiotherapy. Secondary outcomes included post-treatment health-related quality of life, QLQ-HN43 symptom scores, nutritional status, body mass index, weight loss, number of teeth after treatment, change in number of teeth, patient-reported oral symptom burden, functional impact of oral problems, and use of preventive and supportive dental measures.

Exploratory association analyses were performed to examine relationships between xerostomia, dysphagia, taste disturbances, trismus, dental status, nutritional indicators, patient-reported oral burden, and quality-of-life outcomes.

### 2.5. Statistical Analysis

Statistical analysis was performed using JASP version 0.96.0.0. Continuous variables were presented as mean ± standard deviation and median with interquartile range, while categorical variables were presented as frequencies and percentages.

Due to the limited sample size and exploratory design, non-parametric methods were used for inferential analyses. Spearman’s rank correlation coefficient was used to assess associations between continuous or ordinal variables. Mann–Whitney U tests and Fisher’s exact tests were considered for group comparisons when group sizes were sufficient, but the final inferential analysis focused on selected Spearman correlation analyses because of the small sample size and exploratory design.

A *p*-value < 0.05 was considered statistically significant. Findings with *p*-values between 0.05 and 0.10 were interpreted descriptively as trends and not as statistically significant findings. Because of the exploratory nature of the study and the limited sample size, emphasis was placed on effect direction, clinical relevance, and hypothesis generation rather than confirmatory inference.

### 2.6. Ethical Considerations

The study was conducted in accordance with the Declaration of Helsinki. Ethical approval for the part of the study conducted at the University Medical Centre Maribor was obtained from the Medical Ethics Committee of the University Medical Centre Maribor (approval No. UKC-MB-KME-51/25; 1 October 2025). Approval for collaboration and study conduct at the Community Health Centre dr. Adolfa Drolca Maribor was obtained from the Ethics Committee of the Community Health Centre dr. Adolfa Drolca Maribor (approval No. 02/010/03-23/01/25; 21 October 2025).

Patient participation in the questionnaire-based follow-up assessment was voluntary, and informed consent was obtained according to institutional requirements. Clinical data were handled confidentially and analyzed in a pseudonymized form. No directly identifiable patient information was included in the analysis or manuscript.

## 3. Results

### 3.1. Patient, Tumor, Treatment, and Follow-Up Characteristics

The final cohort comprised 20 patients, predominantly men, with a median age of 62.5 years. Oropharyngeal and oral cavity/lip tumors were the most common sites, and most patients had locally advanced disease. Treatment was predominantly multimodal: surgery was performed in 80.0% of patients and chemotherapy was administered in 45.0%. The median radiotherapy dose was 66.0 Gy delivered in 33 fractions, and the median interval between radiotherapy completion and questionnaire assessment was 3.0 months. Detailed patient, tumor, treatment, and follow-up characteristics are presented in Table 1.

### 3.2. Oral, Dental, Functional, and Nutritional Status

Oral and functional complications were frequent, with dysphagia, xerostomia, and taste disturbances recorded in 85.0%, 80.0%, and 75.0% of patients, respectively; objective trismus was present in 40.0%. Periodontitis was identified in 55.0% of patients, all of whom had stage III or IV disease. The reduction in the number of teeth primarily reflected preventive extractions performed during pre-radiotherapy dental preparation and should not be interpreted as radiation-induced tooth loss. Weight loss greater than 10% occurred in 55.0% of patients, while 95.0% used oral nutritional supplements. Detailed oral, dental, functional, and nutritional findings are presented in Table 2.

### 3.3. EORTC Quality-of-Life Outcomes

The mean QLQ-C30 global health status/QoL score was 54.2 ± 17.4. Among the QLQ-C30 functional scales, cognitive and emotional functioning showed the highest scores, whereas role functioning was lowest. Pain, fatigue, appetite loss, and insomnia represented the most prominent general symptom domains. On the QLQ-HN43, the greatest burden involved fear of progression, dry mouth and sticky saliva, problems opening the mouth, social eating, weight loss, body image, and dental problems. Detailed EORTC QLQ-C30 and QLQ-HN43 results are presented in Table 3.

### 3.4. Patient-Reported Oral Symptoms, Functional Impact, and Preventive/Supportive Care

Dry mouth, taste disturbances, chewing difficulties, limited mouth opening, and swallowing difficulties were the most frequently reported oral symptoms. Functional limitations were also common, particularly difficulties eating hard or dry food and soft or liquid food. Although all patients reported pre-radiotherapy dental examination and oral hygiene instruction, the reported use of fluoride, mouth-opening exercises, and regular dental follow-up was lower. Detailed patient-reported symptoms, functional impact, and preventive/supportive care findings are presented in Table 4.

### 3.5. Exploratory Associations Between Oral, Dental, Functional, and Quality-of-Life Outcomes

The strongest association was observed between swallowing problems and social eating difficulties (ρ = 0.798, *p* < 0.001). Greater oral functional impact was associated with more severe problems opening the mouth, while reduced objective mouth opening was associated with greater patient-reported limitation. Better dentition at follow-up was associated with higher global quality of life, whereas greater reduction in tooth number was associated with more dental problems. No significant associations were observed between percentage weight loss and swallowing, social eating, appetite loss, or oral functional impact. Detailed exploratory correlations are presented in Table 5.

Given the small sample size and the number of correlations examined, these findings should be interpreted as exploratory and hypothesis-generating and not as confirmatory evidence.

## 4. Discussion

This exploratory observational study, combining a retrospective record review with a prospective cross-sectional assessment, integrated clinical, dental, nutritional, functional, and patient-reported outcomes after head and neck radiotherapy. The cohort showed a substantial oral-functional burden, particularly involving swallowing, salivary function, taste, mouth opening, dentition, and nutritional status. This overall pattern is consistent with previous evidence linking dysphagia, dysgeusia, oral mucositis, and xerostomia with impaired oral intake, weight loss, and quality of life [44], and with EORTC-based survivorship studies identifying swallowing, social eating, dental problems, mouth opening, dry mouth, and sticky saliva as relevant post-treatment domains [45,46,47]. Given the small sample size and the number of correlations examined, the observed associations should be regarded as exploratory and hypothesis-generating rather than confirmatory and require validation in larger prospective cohorts.

Oral mucositis and radiodermatitis were common during treatment, whereas dysphagia, xerostomia, and taste disturbances remained prominent at follow-up. These findings are consistent with the recognized toxicity profile of head and neck radiotherapy [15]. Jham et al. reported mucositis, candidiasis, and xerostomia during treatment and persistent xerostomia, radiation caries, and osteoradionecrosis after treatment [34]. Differences in xerostomia frequency between studies may reflect sample size, treatment modality, tumor-site distribution, follow-up timing, and the use of both clinical documentation and patient-reported assessment.

Given that oral mucositis was recorded in 90.0% of the present cohort, preventive and supportive management warrants particular attention. Poposki et al. reviewed contemporary preventive modalities, including patient education, professional oral care, benzydamine, photobiomodulation, cryotherapy, mucosal-protective agents, analgesic mouth rinses, and orally administered glutamine [40]. Photobiomodulation is particularly relevant in patients receiving head and neck radiotherapy when established treatment parameters are followed. However, the evidence and indications for other interventions vary, and their use should therefore be individualized according to the treatment regimen, patient characteristics, and clinical context.

The median interval between radiotherapy completion and questionnaire assessment was only 3.0 months. Therefore, the present findings primarily reflect early post-treatment morbidity rather than the full spectrum of late radiation-related complications. Chronic conditions such as radiation-related caries, progressive periodontal deterioration, persistent salivary gland dysfunction, tissue fibrosis, and osteoradionecrosis may develop or become clinically apparent over longer follow-up periods and are likely underrepresented in this cohort. Accordingly, the relatively limited occurrence of late complications in the present study should not be interpreted as evidence of a low long-term risk.

Dry mouth and taste disturbances were among the most burdensome patient-reported symptoms. Salivary dysfunction compromises lubrication, buffering, remineralization, and oral clearance [25], while dysgeusia may impair food intake and quality of life [18,44]. The positive association between swallowing problems and dry mouth/sticky saliva further suggests that xerostomia may contribute to functional swallowing difficulty in addition to oral discomfort.

Dental status requires careful interpretation. The observed reduction in tooth number largely reflected preventive extractions performed before radiotherapy and pre-existing dental compromise rather than post-radiotherapy tooth loss. This distinction is clinically important because pre-treatment dental planning must balance the elimination of oral foci, preservation of functional dentition, and the subsequent risk of osteoradionecrosis [33]. The reduced dental reserve observed in this cohort was also accompanied by advanced periodontal disease, as all patients with periodontitis had stage III or IV disease.

Although radiation-related caries was not assessed as a cause of tooth loss in the present study, long-term dental risk after radiotherapy remains clinically relevant. Moore et al. reported a post-radiotherapy caries incidence of approximately 29% and emphasized intensive oral hygiene instruction, dietary advice, fluoride-based prevention, and regular recall to reduce dental deterioration, post-treatment extractions, osteoradionecrosis risk, and quality-of-life impairment [19]. Accordingly, the change in tooth number in this cohort should be interpreted primarily in the context of pre-radiotherapy dental preparation, while continued post-treatment prevention remains essential.

Functional limitations related to eating were prominent. The strong association between swallowing problems and social eating difficulties supports the interpretation of dysphagia as both a physiological impairment and a broader functional and psychosocial problem. This is consistent with Ninfa et al., who found that social eating was associated with swallowing-related quality of life, swallowing symptoms, nutritional status, and other clinical and psychosocial factors [46].

Reduced mouth opening also contributed to functional burden. Previous evidence has shown substantial variability in the prevalence of trismus after head and neck radiotherapy, depending on treatment technique and study characteristics [22]. In the present cohort, objective mouth opening was inversely associated with patient-reported problems opening the mouth and with oral functional impact, supporting the combined use of objective measurement and patient-reported assessment.

The EORTC findings demonstrated a multidimensional post-treatment burden, with fear of progression, dry mouth and sticky saliva, problems opening the mouth, social eating, weight loss, body image, and dental problems among the most affected domains. However, because baseline EORTC QLQ-C30 and QLQ-HN43 assessments were unavailable, these scores should be interpreted as a cross-sectional description of post-treatment status rather than as evidence of deterioration attributable to radiotherapy.

Comparable EORTC-based studies have identified similar head-and-neck-specific problems after treatment. Wan Leung et al. reported associations involving global quality of life, swallowing, social eating, dental problems, mouth opening, dry mouth, and sticky saliva, although their study used the QLQ-H&N35 rather than the QLQ-HN43 [45]. In a prospective Slovenian cohort, Gradišar et al. found that taste and salivary-related impairments remained worse than baseline 10–12 weeks after radiotherapy [47], supporting the clinical relevance of the corresponding symptoms observed in the present cohort.

Nutritional deterioration was frequent, with more than half of the cohort experiencing weight loss greater than 10%. Dysphagia, dysgeusia, xerostomia, and oral mucositis are recognized nutrition-impact symptoms that may impair appetite and oral intake [44]. However, percentage weight loss was not significantly associated with swallowing, social eating, appetite loss, or oral functional impact in this cohort. These negative findings should be interpreted cautiously because weight loss in head and neck cancer is multifactorial and may also reflect tumor characteristics, treatment intensity, systemic inflammation, baseline nutritional status, nutritional support, and the timing of assessment.

A key strength of this study is the integration of clinical, dental, nutritional, and patient-reported data. The EORTC QLQ-C30 assessed general cancer-related quality of life, the QLQ-HN43 captured head-and-neck-specific symptoms and functional problems, and the focused oral-health questionnaire provided additional information on symptom severity, functional impact, and supportive care. Together, these complementary measures enabled a broader assessment of post-treatment oral-functional burden than any single data source alone.

Despite universal pre-radiotherapy dental assessment and oral hygiene instruction, the reported use of fluoride, mouth-opening exercises, and regular dental follow-up was limited. This pattern may reflect barriers related to access, continuity of care, treatment burden, adherence, or coordination between oncology and dental services. Current Slovenian recommendations and dental-management reviews emphasize fluoride-based prevention, long-term dental follow-up, xerostomia management, trismus prevention, and specialist referral when invasive dental treatment is required after radiotherapy [31,36,37].

In practical terms, survivorship care may benefit from a coordinated multidisciplinary pathway that includes individualized caries-risk assessment, professional plaque control, dietary counseling, fluoride-based prevention, symptom-directed management of xerostomia, serial mouth-opening measurements with supervised exercises, and timely referral for nutritional or swallowing assessment. Patients requiring invasive procedures in irradiated bone should be referred to an appropriate specialist service. These measures should be considered practical clinical recommendations informed by existing guidance and the care gaps observed in this cohort, rather than conclusions regarding their effectiveness derived from the present exploratory study.

This study has several limitations. First, the small sample size (*n* = 20) substantially limited statistical power, increased the uncertainty of the estimates, and prevented robust subgroup analyses. Because the cohort was recruited within a single regional clinical setting, the findings may not be generalizable to broader populations of patients with head and neck cancer or to other healthcare settings. Accordingly, the results should be interpreted as exploratory and hypothesis-generating rather than confirmatory. Second, the cohort was clinically heterogeneous with respect to tumor site, treatment modality, and the interval between radiotherapy completion and questionnaire assessment. These factors may influence the type, severity, and persistence of oral complications and patient-reported quality-of-life outcomes; however, their independent effects could not be evaluated in this small cohort. In addition, the median follow-up interval of 3.0 months means that the study predominantly captured early post-treatment effects, while later complications may have been underrepresented. Third, the combined retrospective record-review design and prospective cross-sectional assessment of patient-reported outcomes precluded evaluation of changes over time and temporal relationships between treatment-related complications and quality-of-life outcomes. Consequently, the observed associations should be interpreted as cross-sectional relationships and cannot establish causality or temporal sequence. Patient-reported outcomes were assessed once at follow-up, and baseline EORTC QLQ-C30 and QLQ-HN43 scores were not available. Fourth, some variables were based on retrospective clinical documentation and patient self-report, which may introduce information and recall bias. Fifth, because of the limited sample size, multivariable analyses were not performed, and potential confounding by tumor site, radiotherapy dose distribution, chemotherapy, surgery, baseline oral status, comorbidities, and nutritional interventions could not be controlled.

Despite these limitations, the study has several strengths. It integrates clinical, dental, nutritional, and patient-reported data in a real-world cohort of patients treated with radiotherapy for head and neck cancer. It includes both validated EORTC instruments and a targeted oral-health questionnaire, allowing multidimensional assessment of oral morbidity and its functional impact. In addition, the analysis focused on clinically interpretable descriptive findings and selected exploratory associations rather than overextended statistical modeling, which is appropriate given the limited sample size.

Future research should prioritize adequately powered, prospective multicenter studies with standardized baseline and serial follow-up assessments. Such studies should include repeated EORTC QLQ-C30 and QLQ-HN43 measurements, longer follow-up to capture both early and late complications, and detailed information on tumor site, treatment modality, radiotherapy dose distribution, baseline oral status, and nutritional interventions. Larger cohorts would also enable adjusted multivariable analyses, clinically meaningful subgroup comparisons, and evaluation of whether structured multidisciplinary dental, nutritional, and functional-care pathways improve patient outcomes.

## 5. Conclusions

In this small exploratory cohort, frequent oral and functional problems were observed at early post-treatment follow-up in patients with head and neck cancer treated with radiotherapy, including xerostomia, dysphagia, taste disturbances, reduced mouth opening, dental problems, and clinically relevant weight loss. The observed associations between swallowing, social eating, mouth opening, dental status, and quality of life should be regarded as hypothesis-generating rather than confirmatory. Because baseline EORTC assessments were unavailable, the patient-reported outcomes describe post-treatment status and do not quantify changes attributable to radiotherapy. These findings suggest the potential value of coordinated dental, nutritional, functional, and patient-reported outcome monitoring during survivorship. Larger prospective multicenter studies with baseline and longitudinal assessments are required to confirm these observations and evaluate structured multidisciplinary care pathways.

## Figures and Tables

**Table 1:** Patient, tumor, treatment, and follow-up characteristics of the study cohort.

Characteristic	Value
**Patient and tumor characteristics**	
Number of patients	20
Age, years	62.5 [IQR 13.3]; range 45–77
***Sex***	
Male	18 (90.0%)
Female	2 (10.0%)
***Tumor site***	
Oral cavity/lip	7 (35.0%)
Oropharynx	8 (40.0%)
Hypopharynx	1 (5.0%)
Larynx	2 (10.0%)
Nasopharynx	1 (5.0%)
Salivary gland	1 (5.0%)
***Histopathology***	
Squamous cell carcinoma	19 (95.0%)
Carcinosarcoma	1 (5.0%)
***TNM T category***	
T0	1 (5.0%)
T1	3 (15.0%)
T2	4 (20.0%)
T3	6 (30.0%)
T4	6 (30.0%)
***TNM N category***	
N0	8 (40.0%)
N1	3 (15.0%)
N2	6 (30.0%)
N3	3 (15.0%)
***TNM M category***	
M0	20 (100.0%)
***HPV status***	
HPV-negative	10 (50.0%)
HPV-positive	10 (50.0%)
***Tumor side***	
Left	9 (45.0%)
Right	11 (55.0%)
**Treatment and follow-up characteristics**	
***Treatment modality***	
Radiotherapy alone	3 (15.0%)
Radiotherapy + chemotherapy	2 (10.0%)
Surgery + radiotherapy	8 (40.0%)
Surgery + radiotherapy + chemotherapy	7 (35.0%)
Radiotherapy dose, Gy	66.0 [IQR 7.0]; range 56–70
Number of RT fractions	33.0 [IQR 5.0]; range 28–35
***Chemotherapy***	
No	11 (55.0%)
Yes	9 (45.0%)
Chemotherapy regimen among patients receiving chemotherapy	*n* = 9
Cisplatin	6 (66.7%)
Cisplatin + carboplatin	2 (22.2%)
Carboplatin	1 (11.1%)
Number of chemotherapy cycles among patients receiving chemotherapy	6.0 [IQR 0.0]; range 5–7
***Surgery***	
No	4 (20.0%)
Yes	16 (80.0%)
Time since RT, months	3.0 [IQR 3.3]; range 1–7

**Note:** Values are presented as *n* (%) or median [IQR]; range, unless otherwise indicated. RT, radiotherapy; chemotherapy, systemic chemotherapy.

**Table 2:** Oral, dental, functional, and nutritional characteristics of the study cohort.

Characteristic	Value
***Oral and functional complications***	
Oral mucositis during RT	18 (90.0%)
Radiodermatitis during RT	18 (90.0%)
Dysphagia after treatment	17 (85.0%)
Xerostomia after treatment	16 (80.0%)
Taste disturbances after treatment	15 (75.0%)
Mouth opening, mm	40.7 ± 13.5; 40.5 [18.8]; range 20–65
Trismus ≤ 35 mm	8 (40.0%)
***Dental status***	
Periodontitis	11 (55.0%)
Periodontitis stage III/IV	11 (55.0%)
Number of teeth before RT	18.6 ± 8.2; 22.5 [10.5]; range 0–30
Number of teeth at follow-up	12.0 ± 8.9; 13.0 [15.0]; range 0–26
Change in number of teeth from pre-RT assessment to follow-up	6.7 ± 5.8; 7.0 [7.5]; range 0–24
Edentulous at follow-up	3 (15.0%)
***Nutritional status***	
Weight before RT, kg	78.4 ± 15.1; 76.0 [12.8]; range 62–130
Weight at follow-up, kg	68.7 ± 12.7; 67.5 [11.3]; range 54–114
Absolute weight loss, kg	9.8 ± 7.0; 8.5 [10.5]; range 0–20
Percentage weight loss, %	12.0 ± 8.1; 11.6 [12.0]; range 0–25
Weight loss > 10%	11 (55.0%)
BMI before RT, kg/m^2^	26.3 ± 4.2; 25.7 [3.0]; range 21.0–39.2
BMI at follow-up, kg/m^2^	23.1 ± 3.6; 22.5 [3.4]; range 17.8–34.4
Use of oral nutritional supplements	19 (95.0%)
Supplements per day among users	1.7 ± 0.6; 2.0 [1.0]; range 1–3

**Note:** Values are presented as *n* (%) or mean ± standard deviation; median [interquartile range]; range. RT, radiotherapy; BMI, body mass index.

**Table 3:** EORTC QLQ-C30 and EORTC QLQ-HN43 outcomes at post-treatment follow-up.

Scale/Domain	Score
***EORTC QLQ-C30***	
***Global health status***	
Global health status/QoL	54.2 ± 17.4; 50.0 [20.9]; range 25.0–83.3
***Functional scales***	
Physical functioning	67.7 ± 20.8; 66.7 [23.4]; range 20.0–100.0
Role functioning	47.5 ± 31.7; 50.0 [33.4]; range 0.0–100.0
Emotional functioning	77.9 ± 20.5; 79.2 [27.1]; range 33.3–100.0
Cognitive functioning	85.8 ± 18.2; 83.3 [16.7]; range 33.3–100.0
Social functioning	70.8 ± 20.9; 66.7 [20.8]; range 33.3–100.0
***Symptom scales/items***	
Fatigue	41.1 ± 28.0; 33.3 [25.1]; range 0.0–100.0
Nausea and vomiting	11.7 ± 25.4; 0.0 [4.2]; range 0.0–100.0
Pain	42.5 ± 25.6; 50.0 [20.9]; range 0.0–100.0
Dyspnea	11.7 ± 19.6; 0.0 [33.3]; range 0.0–66.7
Insomnia	30.0 ± 37.3; 16.7 [41.7]; range 0.0–100.0
Appetite loss	33.3 ± 35.9; 33.3 [41.7]; range 0.0–100.0
Constipation	20.0 ± 29.4; 0.0 [33.3]; range 0.0–100.0
Diarrhea	1.7 ± 7.4; 0.0 [0.0]; range 0.0–33.3
Financial difficulties	16.7 ± 22.9; 0.0 [33.3]; range 0.0–66.7
***EORTC QLQ-HN43***	
***Head-and-neck-specific symptom and functional domains***	
Pain in the mouth	14.2 ± 13.5; 8.3 [25.0]; range 0.0–41.7
Swallowing	25.8 ± 25.8; 25.0 [41.7]; range 0.0–91.7
Problems with teeth	31.1 ± 24.9; 27.8 [16.7]; range 0.0–100.0
Dry mouth and sticky saliva	46.7 ± 23.9; 50.0 [33.4]; range 0.0–100.0
Problems with senses	27.5 ± 30.2; 25.0 [37.5]; range 0.0–100.0
Speech	26.3 ± 22.1; 23.4 [35.0]; range 0.0–80.0
Body image	31.1 ± 32.8; 22.2 [50.0]; range 0.0–100.0
Social eating	33.3 ± 31.1; 25.0 [60.5]; range 0.0–91.7
Sexuality	24.2 ± 29.9; 16.7 [33.3]; range 0.0–100.0
Shoulder problems	25.8 ± 32.2; 8.4 [37.5]; range 0.0–100.0
Skin problems	19.4 ± 18.7; 11.1 [25.0]; range 0.0–66.7
Fear of progression	52.5 ± 28.2; 41.7 [33.4]; range 16.7–100.0
Problems opening the mouth	40.0 ± 36.8; 33.3 [66.7]; range 0.0–100.0
Coughing	13.3 ± 25.1; 0.0 [33.3]; range 0.0–100.0
Social contact	20.0 ± 31.3; 0.0 [33.3]; range 0.0–100.0
Swelling in the neck	21.7 ± 29.2; 0.0 [33.3]; range 0.0–100.0
Weight loss	31.7 ± 33.3; 33.3 [41.6]; range 0.0–100.0
Wound healing problems	20.0 ± 31.4; 0.0 [33.3]; range 0.0–100.0
Neurological problems	28.3 ± 34.7; 0.0 [66.7]; range 0.0–100.0

**Note:** All EORTC scores were transformed to a 0–100 scale. For QLQ-C30 global health status and functional scales, higher scores indicate better quality of life or better functioning, whereas higher symptom scores indicate greater symptom burden. For QLQ-HN43, higher scores indicate greater symptom burden or more problems. Values are presented as mean ± standard deviation; median [interquartile range]; range. EORTC, European Organisation for Research and Treatment of Cancer; QoL, quality of life; SD, standard deviation.

**Table 4:** Patient-reported oral symptoms, functional impact, and preventive/supportive dental care after treatment.

Patient-Reported Oral Symptom	Any Occurrence, *n* (%)	Quite a Bit/Very Much, *n* (%)
Dry mouth	17 (85.0%)	14 (70.0%)
Oral pain	9 (45.0%)	7 (35.0%)
Burning or irritating sensation	11 (55.0%)	10 (50.0%)
Chewing difficulties	14 (70.0%)	14 (70.0%)
Swallowing difficulties	13 (65.0%)	13 (65.0%)
Taste disturbances	15 (75.0%)	13 (65.0%)
Limited mouth opening	14 (70.0%)	12 (60.0%)
Ulcers or mucosal inflammation	9 (45.0%)	6 (30.0%)
Oral fungal infection	3 (15.0%)	1 (5.0%)
Oral symptom count	5.3 ± 2.7; 5.5 [4.5]; range 1–9	—
Severe symptom count	4.5 ± 2.6; 4.5 [4.0]; range 0–9	—
**Functional impact**		
***Functional impact item***	**Quite a bit/very much, *n* (%)**	
Difficulties eating hard or dry food	16 (80.0%)	
Difficulties eating soft or liquid food	16 (80.0%)	
Reduced food intake	13 (65.0%)	
Difficult oral hygiene	4 (20.0%)	
Functional impact count	2.5 ± 1.2; 3.0 [1.0]; range 0–4	
**Preventive/supportive dental care**		
***Preventive/supportive dental care item***	**Yes, *n* (%)**	
Dental examination before RT	20 (100.0%)	
Oral hygiene instructions	20 (100.0%)	
Fluoride use	11 (55.0%)	
Products for dry mouth	14 (70.0%)	
Mouth-opening exercises	6 (30.0%)	
Regular dental follow-up	7 (35.0%)	
Adequate information received	17 (85.0%)	
Difficult access to dental care	5 (25.0%)	
Preventive/supportive care score	4.8 ± 1.1; 5.0 [2.0]; range 3–6	

**Note:** Values are presented as *n* (%) or mean ± standard deviation; median [interquartile range]; range. RT, radiotherapy. “Quite a bit/very much” indicates clinically relevant symptom severity or functional impact. Percentages are calculated for the total cohort, *n* = 20.

**Table 5:** Selected exploratory Spearman correlations between oral, dental, nutritional, and quality-of-life outcomes.

Association	Spearman’s ρ	*p*-Value
***Oral and functional symptom burden***		
HN43 swallowing score vs. HN43 social eating score	0.798	<0.001
HN43 swallowing score vs. HN43 dry mouth/sticky saliva score	0.623	0.003
HN43 swallowing score vs. HN43 teeth score	0.481	0.032
HN43 social eating score vs. HN43 teeth score	0.629	0.003
HN43 problems opening the mouth vs. HN43 teeth score	0.621	0.003
HN43 dry mouth/sticky saliva score vs. HN43 problems opening the mouth	0.451	0.046
Oral functional impact count vs. oral symptom count	0.583	0.007
Oral functional impact count vs. HN43 problems opening the mouth	0.696	<0.001
***Mouth opening and functional limitation***		
Mouth opening, mm vs. HN43 problems opening the mouth	−0.610	0.004
Mouth opening, mm vs. oral functional impact count	−0.499	0.025
***Dental status and quality of life***		
Number of teeth at follow-up vs. QLQ-C30 global health status/QoL	0.557	0.011
Change in number of teeth from pre-RT assessment to follow-up vs. HN43 teeth score	0.494	0.027
***Nutritional associations***		
HN43 swallowing score vs. percentage weight loss	0.145	0.543
HN43 social eating score vs. percentage weight loss	0.203	0.390
QLQ-C30 appetite loss vs. percentage weight loss	0.213	0.368
Oral functional impact count vs. percentage weight loss	0.192	0.418

**Note:** Spearman’s rank correlation coefficient was used. For QLQ-C30 global health status/QoL, higher scores indicate better quality of life. For QLQ-C30 symptom scales and QLQ-HN43 scales, higher scores indicate greater symptom burden. Higher oral functional impact count indicates a greater number of oral problems with clinically relevant functional impact. RT, radiotherapy; QoL, quality of life.

## Data Availability

The data presented in this study are available from the corresponding author upon reasonable request and subject to institutional and ethical restrictions.

## Abbreviations

BMI, body mass index; EORTC, European Organisation for Research and Treatment of Cancer; HNC, head and neck cancer; HPV, human papillomavirus; IQR, interquartile range; QLQ-C30, Quality of Life Questionnaire Core 30; QLQ-HN43, Quality of Life Questionnaire Head and Neck Cancer Module 43; QoL, quality of life; RT, radiotherapy; SD, standard deviation.
